# Protein co-translocational unfolding depends on the direction of pulling

**DOI:** 10.1038/ncomms5841

**Published:** 2014-09-08

**Authors:** David Rodriguez-Larrea, Hagan Bayley

**Affiliations:** 1 Department of Chemistry, University of Oxford, Oxford OX1 3TA, UK

## Abstract

Protein unfolding and translocation through pores occurs during trafficking between organelles, protein degradation and bacterial toxin delivery. *In vivo*, co-translocational unfolding can be affected by the end of the polypeptide that is threaded into the pore first. Recently, we have shown that co-translocational unfolding can be followed in a model system at the single-molecule level, thereby unravelling molecular steps and their kinetics. Here, we show that the unfolding kinetics of the model substrate thioredoxin, when pulled through an α-haemolysin pore, differ markedly depending on whether the process is initiated from the C terminus or the N terminus. Further, when thioredoxin is pulled from the N terminus, the unfolding pathway bifurcates: some molecules finish unfolding quickly, while others finish ~100 times slower. Our findings have important implications for the understanding of biological unfolding mechanisms and in the application of nanopore technology for the detection of proteins and their modifications.

## Abstract (web summary)

Protein unfolding and translocation through membrane pores occurs in several biological processes and has implications in nanopore technologies. Here, the authors show that the kinetics of unfolding differ depending on which end of the chain enters the pore first.

## Graphical abstract

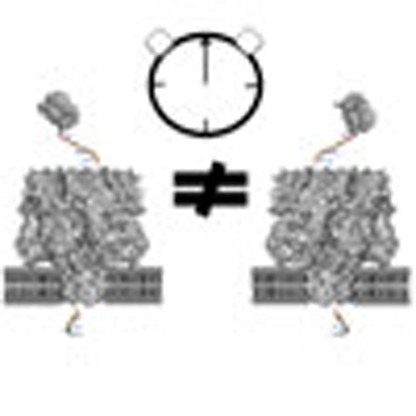

In the course of protein trafficking between organelles, protein degradation and bacterial toxin delivery, proteins are unfolded and translocated through nanometre-sized pores^1^,^2^,^3^,^4^. Early studies on protein degradation mediated by unfoldases revealed that a protein could be unfolded from either end and that the two processes occurred at different rates^4^,^5^,^6^. Furthermore, studies on mitochondrial import rates^1^ found that the unfolding rate depends on which terminus of the protein leads the process. Therefore, neither the molecular pathway(s) nor the kinetics of co-translocational unfolding can be elucidated by force spectroscopy, when simple two-end pulling is used. In a later study, the thermodynamic stability of a translocated protein was correlated with the degradation rate^7^. However, thermodynamic values obtained from temperature or chemical denaturation in bulk solution must be used cautiously when considering the kinetics of force-driven processes, a caveat supported by molecular dynamics simulations^8^,^9^,^10^. Fortunately, recent developments in nanopore technology have allowed the unfolding and translocation of proteins through biological pores to be observed in model systems with the fine detail provided by single-molecule analysis^11^,^12^. Remarkably, by this means, an intermediate step in the co-translocational unfolding of thioredoxin (Trx) pulled from the carboxyl terminus (C terminus) has been detected^11^.

Here, we use this new approach to determine whether and how co-translocational unfolding led by the amino terminus (N terminus) of Trx differs from that led by the C terminus. We find that the unfolding pathways differ; the initial unfolding of the protein is determined by the structure of the region that leads translocation. Unfolding of the remainder of the protein also differs. For example, the pathway for N terminus-first translocation is bifurcated, with a fast and a slow branch. These results have important implications for understanding the mechanisms and evolution of biological co-translocational unfolding and, more practically, for the development of nanopore-based proteomic analysis.

## Results

### C and N termini initiate different unfolding kinetics

**Figure 1:**
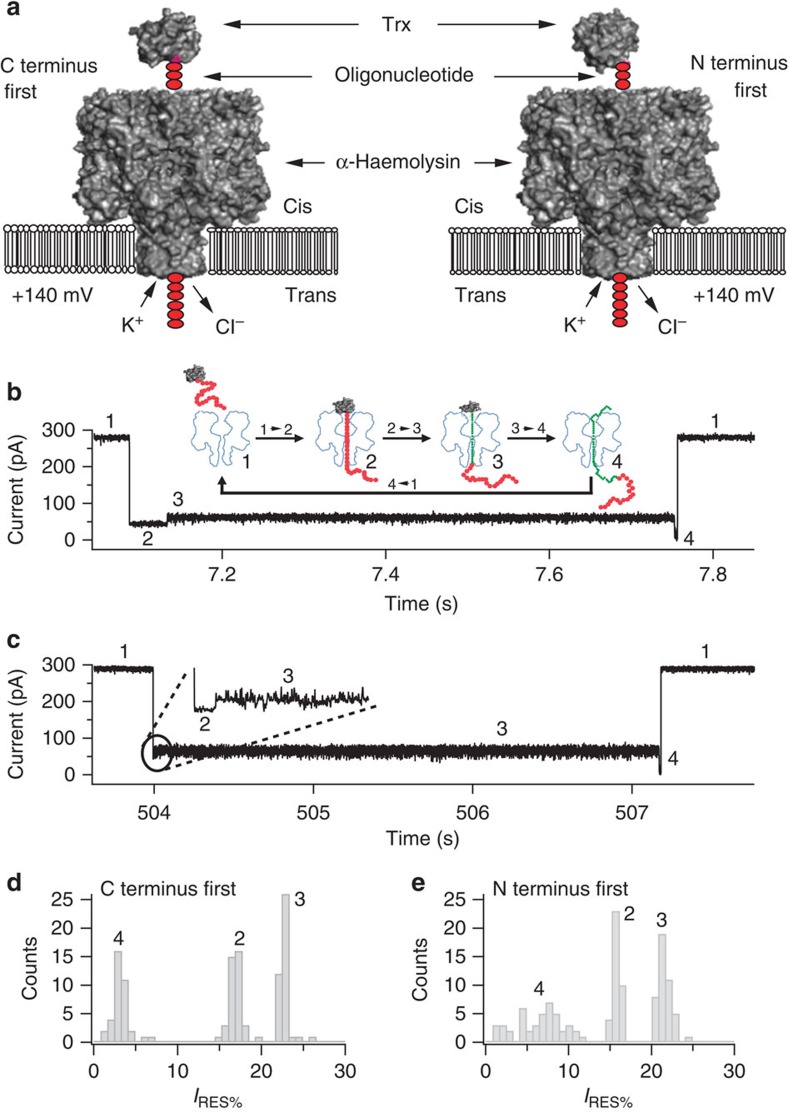
Co-translocational protein unfolding of Trx is a four-step process. (**a**) C terminus threading of Trx V5-C109-oligo(dC)_30_ and N terminus threading of Trx oligo(dC)_30_-S1C-V5 into the cis entrance of the αHL pore. (**b**) Representative current trace showing the four current levels that reflect the co-translocational unfolding of Trx V5-C109-oligo(dC)_30_ at +140 mV in 2 M KCl. (**c**) Representative current trace showing the four current levels that reflect the co-translocational unfolding of Trx oligo(dC)_30_-S1C-V5 at +140 mV in 2 M KCl. (**d**) Event histogram of the residual current levels expressed as *I*_RES%_ at +140 mV observed during the co-translocational unfolding of Trx V5-C109-oligo(dC)_30_ (ref. 11). (**e**) Event histogram of *I*_RES%_ at +140 mV observed during the co-translocational unfolding of Trx oligo(dC)_30_-S1C-V5 (ref. 11). In the case of C terminus pulling, 93% of the observed blockades were of the four-step form. In the case of N-terminal pulling, 91% of the events showed four steps.

Trx V5 (C32S/C35S/A22P/I23V/P68A) was pulled through the α-haemolysin (αHL) pore by using a DNA oligonucleotide leader attached either to an N-terminal cysteine (oligo(dC)_30_-S1C-TrxV5) or to a C-terminal cysteine (TrxV5-C109-oligo(dC)_30_) (ref. 11) (Fig. 1a). The interactions of the Trx constructs with the pore produced series of partial ionic current blockades, which had similarities in the two cases. Both signals comprised three levels with similar residual current (*I*_RES%_) values and the current steps proceeded without reversals (Fig. 1b,c). The *I*_RES%_ values depend on which part of the polymer is within the pore and its conformation (Fig. 1d,e)^13^. Therefore, as demonstrated for C terminus pulling, we suggest that in agreement with the data presented below, in both cases, level 2 represents a state with the DNA leader threaded into the pore, level 3 represents a state after partial unfolding with the polypeptide chain within the pore and level 4 a state during which, after the completion of unfolding, the remainder of the polypeptide diffuses through the pore^11^. The main differences between the two constructs are found in the dwell times of levels 2 and 3, which are discussed below.

**Figure 2:**
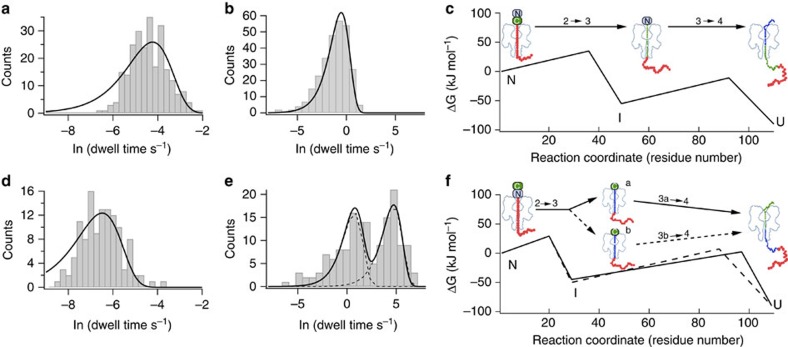
Lifetimes of level 3 for N terminus-first translocation. (**a**) Event histogram of the dwell times in level 2 (step 2→3) for Trx V5-C109-oligo(dC)_30_ at +140 mV (ref. 11). The fit is to a single exponential function and yields a rate constant k_2→3_=70 s^−1^. (**b**) Event histogram of the dwell times in level 3 (step 3→4) for Trx V5-C109-oligo(dC)_30_ at +140 mV (ref. 11). The fit is to a single exponential function and yields a rate constant k_3→4_=1.7 s^−1^. (**c**) Free energy profile of the unfolding steps (2→3 and 3→4) for C terminus-first translocation at +140 mV. A pre-exponential factor of 10^−8^ s^−1^, and ΔG(N→U)=−90 kJ mol^−1^ and ΔG(N→I)=−55 kJ mol^−1^ were assumed, where: N, folded protein; U, unfolded protein; I, unfolding intermediate. (**d**) Event histogram of the dwell times in level 2 (step 2→3) for Trx oligo(dC)_30_-S1C-V5 at +140 mV. The fit is to a single exponential function and yields a rate constant k_3→4_=690 s^−1^. (**e**) Event histogram of the dwell times in level 3 (step 3→4) for Trx oligo(dC)_30_-S1C-V5 at +140 mV. The fit is to a double exponential function and yields rate constants k_3a→4_=0.47 s^−1^ and k_3b→4_=0.0087, s^−1^. The dashed line is the deconvolution into single exponentials. (**f**) Free energy profile of the unfolding steps (2→3 and 3→4) for N terminus-first translocation at +140 mV. A pre-exponential factor of 10^−8^ s^−1^, and ΔG(N→U)=−90 kJ mol^−1^, ΔG(N→I_a_)=−45 kJ mol^−1^ and ΔG(N→I_b_)=−50 kJ mol^−1^ were assumed, where: *I*_a_ and *I*_b_ are the two unfolding intermediates. Data for C terminus first are from ref. 11. Data were collected on six different pores in the case of C terminus first and on 17 different pores in the case of N terminus first.

The transition between level 2 and level 3 (step 2→3) has a strong voltage dependence in both constructs (Supplementary Fig. 1), consistent with the DNA leader being threaded within the αHL pore during level 2 and pulling on the protein to unfold it. The DNA experiences a force (~10 pN at +100 mV (refs 14, 15, 16)) arising from the electric field across the barrel of the pore. Interestingly, step 2→3 is 10–20 times faster (at +140 and +100 mV, respectively) when the protein is pulled from the N terminus as compared with pulling from the C terminus (Table 1), but C terminus pulling shows a steeper voltage dependence (Table 1, Supplementary Fig. 1). Therefore, the nature of the initial unfolding step depends on the direction of pulling and likely involves different parts of the structure of Trx, most probably the regions near the pulling point. Steps 3→4 and 4→1 showed negligible voltage dependence in either construct, which indicates that the DNA leader has already traversed the pore in levels 3 and 4 (Table 1 and Supplementary Fig. 1). A minimal voltage dependence is expected in levels 3 and 4 because the threaded polypeptide chain presents a low density, heterogeneous distribution of both positive and negative charge. Nevertheless, step 3→4 differed in the case of N terminus pulling; the dwell-time distribution in level 3 was found to be bimodal (Fig. 2 and Table 1). Therefore, unfolding initiated from the N terminus can proceed through alternative routes in contrast to the case with C terminus pulling.

Step 4→1, during which the unfolded protein translocates through the pore, most likely by diffusion, might be affected by several factors, which include the direction of motion of the unfolded chain (which can produce different interactions with the wall of the pore; such a phenomenon has been noted for single-stranded DNA^17^), the presence of residual structure at the entrance of the pore and the partial refolding of the chain before substrate translocation is complete. Nevertheless, in the present case, the kinetics of step 4→1 were similar whether translocation was led by the N terminus or the C terminus.

### Pulling from the N terminus unfolds the N terminus

**Figure 3:**
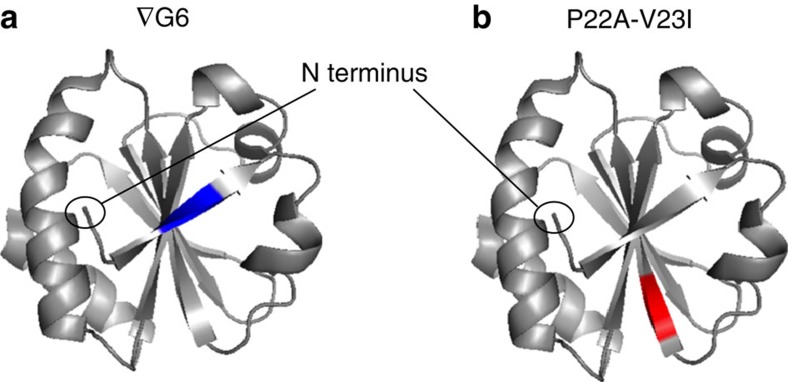
Trx mutants used to study N terminus initiated unfolding. (**a**) In ∇G6, a Gly residue is inserted between residues 5 and 6, which are marked in blue in a molecular model of Trx V5. (**b**) In Trx P22A/V23I, Pro-22 is mutated to Ala and Val-23 to Ile. Residues 22 and 23 are marked in red in a molecular model of Trx V5.

For C terminus-first pulling, it has been already reported that mutations that destabilize structural elements near the C terminus produce an increase in the rate of the first unfolding event (step 2→3), with minor effects on the subsequent steps^11^. Therefore, in this case, the first unfolding event must involve the C terminus of Trx. To further examine step 2→3 for N terminus-first pulling, a Gly residue was inserted between residues 5 and 6 in the first β strand to disrupt the beginning of the central five-stranded sheet (Trx ∇G6, Fig. 3a). We confirmed the destabilizing impact of this mutation on the TrxV5 structure by using circular dichroism to measure unfolding in urea (Supplementary Fig. 2). For the Trx∇G6, we obtained C_1/2,___∇___G6_=6.4±0.4 M and m_1/2,___∇___G6_=2.5±0.9  kJ mol^−1^ M^−1^, while Trx S1C-V5 gave C_1/2,S1C-V5_=9.2±0.8 M and m_1/2,S1C-V5_=3.5±1.3 kJ mol^−1^ M^−1^. Such an effect on the thermodynamic stability of the protein is likely to be reflected in the kinetics of unfolding^18^. Indeed, in the case of ∇G6, step 2→3 could not be detected even at a lower applied potential (+100 mV). Therefore, we suggest step 2→3 for ∇G6 is accelerated to above the detection limit, although we cannot exclude other possibilities. The N-terminal segment may be unfolded before engagement with the pore or it may follow an alternative, faster, pathway. Nevertheless, this result clearly associates the unfolding of the N terminus with step 2→3. As expected, the other steps in co-translocational unfolding were unaffected in ∇G6 (Table 1).

Further, we studied the effect of the mutation P22A-V23I under N terminus pulling (Fig. 3b, Supplementary Fig. 3). P22A-V23I has a strong destabilizing effect on Trx as observed by thermal denaturation and, in the case of C terminus-first pulling, accelerates step 3→4 (ref. 11). Nevertheless, this mutation had little effect on the rates of steps 2→3 and 3→4 when Trx was unfolded from the N terminus (Table 1). Therefore, after entry led by the N terminus, residues 22 and 23 cannot contribute strongly to the transition states of steps 2→3 and 3→4 and must lose their interactions with the remaining folded protein between levels 2 and 3.

Our results show that the co-translocational unfolding kinetics of a model protein depend on the terminus to which the force is applied. Mutants that destabilize the end from which the protein is pulled cause an increase in the initial unfolding rate (step 2→3). When the destabilizing mutation is located far into the polypeptide sequence, an effect on the initial unfolding step is not observed. Therefore, in a first step, the protein denatures locally at a rate that depends on the applied force. This local unfolding affects a limited region of the protein near the point where the force is applied. In other words, in the case of Trx, the mechanical stability of the structure near the N terminus is independent of the mechanical stability of the structure near the C terminus, regardless of the pulling direction. These independent stabilities may mimic important phenomena in biology. For example, in the secretion of an autotransporter protein, which is a directional process, the relative stabilities of C-terminal and N-terminal domains affect the secretion efficiency^19^.

### Molecular model for co-translocational unfolding

**Figure 4:**
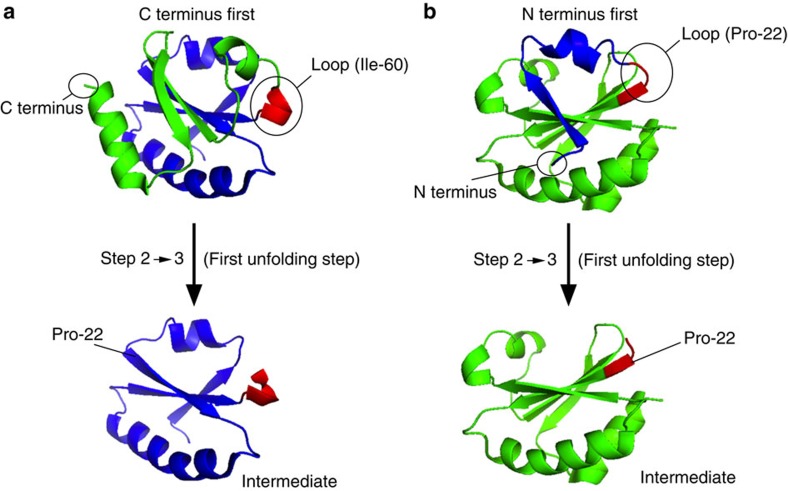
Modular nature of the mechanical resistance to translocation. (**a**) Molecular representation of step 2→3 when the process is initiated from the C terminus. The first unfolding event involves a segment of ~40 amino acids (green) that stretches from the C terminus to a loop at residue 60 (red). The proposed unfolding intermediate (that is the remaining unfolded protein) associated with level 3 is in blue^11^. (**b**) Molecular representation of step 2→3 when the process is initiated from the N terminus. The first unfolding event involves a segment of ~20 amino acids (blue) that stretches from the N terminus to the loop at residue 22. The proposed unfolding intermediate associated with level 3 is in green.

To gain further insight into the extent of unfolding involved in each step, we analysed the effects of urea to obtain kinetic m-values from the slopes of plots of ln k versus [urea] (Table 1, Supplementary Fig. 4)^20^,^21^. Kinetic m-values are related to the difference in accessible surface area between a native or intermediate state and the adjacent transition state. Because this is in turn related to the number of residues newly exposed in the transition state, a minimum estimate of the extent of unfolding in the step in question can be made^20^,^21^. We have already reported that the two unfolding steps that occur when the process is initiated through the C terminus involve 36±8 C-terminal residues in the transition state of step 2→3 and then 43±5 N-terminal residues in the transition state of step 3→4 (Fig. 4a)^11^. We obtained kinetic m-values for N terminus-first co-translocational unfolding: m^≠^_2→3_=0.64±0.2 kJ mol^−1^ M^−1^, m^≠^_3a→6_=2.58±0.67 kJ mol^−1^ M^−1^ and m^≠^_3b→d_=2.27±0.1 kJ mol^−1^ M^−1^ (Supplementary Fig. 4). These values implicate 20±6 N-terminal residues in the transition state of the initial unfolding step (2→3), followed by 77±20 residues in the C terminus unfolding step (3→4) if the protein follows the fast unfolding pathway or 68±3 residues if it follows the slow unfolding pathway. The separately unfolded regions are separated in the Trx structure by a loop that contains Pro-22 and Val-23 (Figs 2f and 4b).

We propose that co-translocational protein unfolding in our model system proceeds by distributing the applied force between the pulling point and a mechanically stable element in the Trx structure. By inspection of the three-dimensional structure, it can be seen that, if Trx is pulled from the N terminus, residues 1–20 can be released first by unzipping a β strand from the edge of the β sheet and then detaching a short helix (Fig. 4b). After residues 21 and 22, which are found in a loop, unfolding must proceed by removal of a central strand from the remaining β sheet, which would offer a high mechanical resistance to unfolding^22^. Similar reasoning can be applied to unfolding led by the C terminus. In this case, the release of residues 109 to 60 must occur by release of the C-terminal α helix followed by unzipping of a β strand. At this point, a loop intervenes and further unfolding proceeds by removal of a central strand from the remaining β sheet (Fig. 4a). According to this view, unfolding from the N terminus involves first the unfolding of residues 1–22, followed by residues 22–109 in a distinct step. Unfolding from the C terminus involves the unfolding of residues 109 to 60, followed by residues 60 to 1. These mechanisms are consistent with the kinetic m-values, which strictly apply to transition states. In addition, the slope of applied potential (force) versus ln(k_2→3_) for C terminus pulling is twice that observed for N terminus pulling, which implies that the distance to the transition state is doubled^23^. The proposed mechanisms could also explain why the P22A-V23I mutation has no effect on unfolding led by the N terminus. After step 2→3, these residues are in a loop that separates the unfolded segment from the remaining folded structure, and the loop unfolds before step 3→4.

## Discussion

Our results show that the unfolding rate of a translocated protein is dependent on the local structure presented to the pore rather than on the global stability of the protein. Consistent with our findings, computer simulations have predicted that the kinetics of N terminus-first and C terminus-first co-translocational unfolding can differ^8^,^9^,^10^. These studies also predicted the existence of intermediates, even for small proteins, and kinetic traps (intermediates with very long unfolding times)^8^,^9^,^10^. Several ensemble experiments involving degradation by the proteasome and import into mitochondria have shown that the rates of these processes depend on which end of the substrate protein is threaded first^1^,^4^. Finally, while we use a model system, the forces^14^,^15^,^16^ applied are similar to those determined for a number of biological processes such as proteasome-catalysed unfolding (~20 pN) (ref. 24), the delivery of bacterial toxins into the cytoplasm (2.7–27 pN) (ref. 3) and co-translocational unfolding during mitochondrial import (11–16 pN) (ref. 25). The internal diameter of the αHL pore is also comparable to the diameter of the pores that mediate these processes^26^,^27^,^28^,^29^. To obtain a complete picture of co-translocational unfolding, it remains necessary to explore how the kinetics vary with different pore sizes and surface chemistries as well as with protein substrates with different topologies. Because, the stability of a protein can depend on which end is pulled into a pore, we might expect that this property has been subjected to strong evolutionary pressure, giving rise to proteins with stabilities towards directional pulling that fit their functions^4^,^19^.

## Methods

### αHL pores

Wild-type monomers of αHL were expressed in an E. coli *in vitro* transcription/translation system and oligomerized into heptameric pores by incubation with rabbit red blood cell membranes^30^. The heptameric αHL pores were purified by SDS-polyacrylamide gel electrophoresis^30^. αHL pores extracted from the gel (0.2 μl, ~1 ng μl^−1^) were added to the cis compartment of a bilayer apparatus. After a single pore had inserted the cis compartment was manually perfused with fresh buffer to prevent further insertions^31^.

### Trx and oligonucleotide-Trx conjugates

The Trx V5 gene^11^ was cloned into the pET 30a+ plasmid (TopGene). Ser-1 was mutated to Cys by using a Quick Change II XL site-directed mutagenesis kit (Stratagene). The mutation was verified by DNA sequencing. The plasmid was transformed into *E. coli* BL21(DE3) cells (Novagen), and Trx was produced by isopropyl-β-D-1-thiogalactopyranoside induction during the exponential growth phase in 0.25 l of LB medium. The expressed Trx was purified by DNA precipitation with streptomycin sulphate, followed by size-exclusion chromatography (Superdex 75 10/300, GE Healthcare) and anion exchange (HiTrap Q HP, GE Healthcare).

A 5′-thiol (hexamethylene linker)-modified oligo(dC)_30_ (Integrated DNA Technologies) was activated with 2,2′-dipyridyl disulphide^32^. After reduction for 24 h with 10 mM DTT, followed by removal of the DTT by size-exclusion chromatography (PD10 column, GE Healthcare), a cysteine mutant of Trx was reacted with the activated oligonucleotide for 16 h at room temperature^32^. The product was purified by ion exchange chromatography on a HiTrap Q FF column (GE Healthcare) with a gradient of 0 to 1 M KCl in 10 mM Tris.HCl, 1 mM EDTA, pH 8.0. After SDS-polyacrylamide gel electrophoresis, the peak containing the conjugate stained both for protein and DNA. The mass of the conjugate, oligo(dC)_30_-S1C-V5-P22A-V23I, was verified by ESI-MS. The concentration of the protein conjugate was determined from the calculated molar extinction coefficient of oligo(dC)_30_ at 260 nm.

### Electrical measurements and data analysis

Planar lipid bilayers recordings were conducted at 22.0±1.5 °C. A bilayer of 1,2-diphytanoyl-*sn*-glycero-3-phosphocholine (Avanti Polar Lipids) was made across an aperture with a diameter of ~100 μm in a Teflon film (Goodfellow), which separated two compartments of 1 ml each (cis and trans)^31^. The electrolyte solution was 10 mM HEPES, 2 M KCl, pH 7.2. For the experiments with urea, we used the same buffer prepared with the desired concentration of urea. Following the insertion of a single αHL pore, the cis compartment was perfused by manual pipetting to remove excess αHL. Ag/AgCl electrodes connected to a patch-clamp amplifier (Axopatch 200B, Axon Instruments) were used to measure the ionic current through the αHL pore. The amplified signal was filtered at 5 kHz and data were collected at 20 kHz with a Digidata 1440A digitizer (Axon Instruments). Data were analysed with pClamp software (Molecular Devices). Data for C terminus-first translocation were obtained from ref. 11. For the N terminus-first experiments, 91% of the events showed the characteristic four-level signal and of these 60% that did not show a noisy level 3 were processed (Supplementary Figs 5 and 6). In the present work, we used 62 pores and each experimental condition was repeated at least three times. Residual current (*I*_RES%_) values were determined for each level (*I*_RES%_=*I*_B_/*I*_O_ × 100, where *I*_B_ is the mean current in level 2, 3 or 4 and *I*_O_ is the mean current through the open pore). Dwell-time distributions of each level were analysed in Igor Pro 6.12A (WaveMetrics), by using in each case the data compiled from three or more pores. Errors for oligo(dC)_30_-S1C-TrxV5 translocation kinetics reflect the coefficient confidence bounds (1σ) of fits of event duration histograms to single exponentials (double exponentials for step 3→4). To obtain kinetic m-values, rate constants were first measured in experiments with 0, 1 and 2 M urea at +100, +120 and +140 mV. Each applied potential gave a kinetic m-value and the mean value was taken, which assumes that that the transition state structure is not voltage dependent over the range explored.

## Author contributions

D.R.-L. planned research, performed experiments, analysed data and wrote the paper. H.B. planned the research and wrote the paper.

## Additional information

**How to cite this article**: Rodriguez-Larrea, D. and Bayley, H. Protein co-translocational unfolding depends on the direction of pulling. *Nat. Commun.* 5:4841 doi: 10.1038/ncomms5841 (2014).

## Supplementary Material

Supplementary Information Supplementary Figures 1-6 and Supplementary Reference

## Figures and Tables

**Table 1:** Rates of co-translocational unfolding and translocation.

**Trx construct**	**Rate step 2**→**3**	**Rate step 3**→**4**	**Rate step 4**→**1**
oligo(dC)_30_-S1C-V5, +100 mV	140±10 s^−1^	0.47±0.03 s^−1^0.0058±0.0002, s^−1^	220±20 s^−1^
oligo(dC)_30_-S1C-V5, +140 mV	690±10 s^−1^	0.47±0.2 s^−1^0.0087±0.0003, s^−1^	120±20 s^−1^
V5-C109-oligo(dC)_30_, +100 mV	6±1 s^−1^	1.5±0.1 s^−1^	100±30 s^−1^
V5-C109-oligo(dC)_30_, +140 mV	70±20 s^−1^	1.7±0.1 s^−1^	80±20 s^−1^
oligo(dC)_30_-S1C-V5, ∇6G, +140 mV	Not detected	0.62±0.05 s^−1^0.0075±0.0005, s^−1^	210±10 s^−1^
oligo(dC)_30_-S1C-V5, P22A-V23I, +140 mV	800±70 s^−1^	0.8±0.2 s^−1^0.0084±0.0004, s^−1^	190±20 s^−1^
oligo(dC)_30_-S1C-V5, 2 M urea, +140 mV	1,000±100 s^−1^	7±1 s^−1^0.051±0.003 s^−1^	200±10 s^−1^

Trx, thioredoxin.

Values for Trx oligo(dC)_30_-S1C-V5 (N terminus first) were derived from exponential or biexponential fits to dwell-time histograms, in which the data from at least three independent experiments were compiled. Errors for N terminus-first pulling represent the coefficient confidence bounds (1σ) obtained in the fits. Values for Trx V5-C109-oligo(dC)_30_ (C terminus first) were obtained from ref. 11. Errors represent the s.d. from independent experiments (*n*=6).
