# Supplementary material for: Protein co-translocational unfolding depends on the direction of pulling
Source: Nat Commun. 2014 Sep 8;5:4841. doi: 10.1038/ncomms5841 (PMC4164780; doi:10.1038/ncomms5841)
Supplement: Supplementary Information — Supplementary Figures 1-6 and Supplementary Reference [file ncomms5841-s1.pdf]

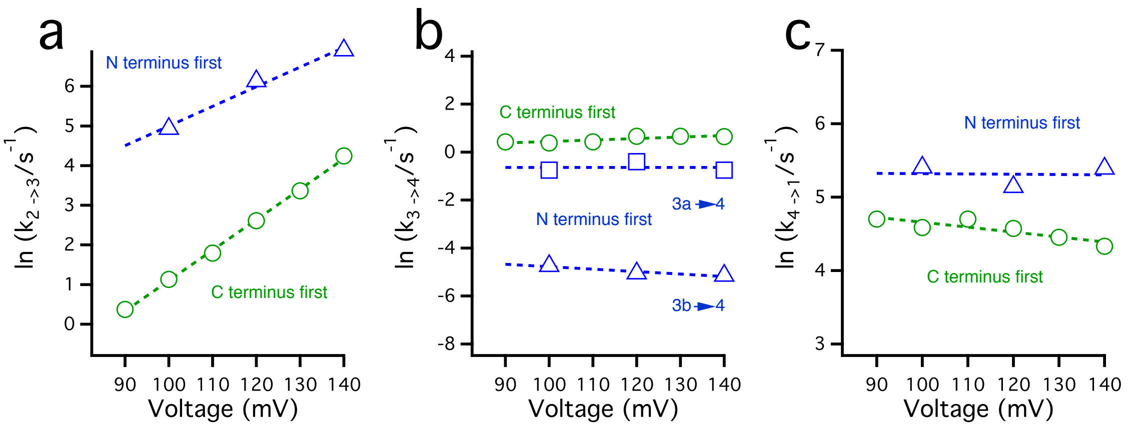

Supplementary Figure 1: Voltage dependence of the co-translocational unfolding of Trx V5-C109-oligo(dC)$_{30}$ (C-terminus first)[1] and Trx oligo(dC)$_{30}$-C1S-V5 (N-terminus first) within the αHL pore. (**a**) Voltage dependence of the rate of step 2→3: (O) V5-C109-oligo(dC)$_{30}$; (Δ) oligo(dC)$_{30}$-C1S-V5. (**b**) Voltage dependence of the rate of step 3→4: (O) V5-C109-oligo(dC)$_{30}$; (□) oligo(dC)$_{30}$-C1S-V5, fast pathway; (Δ) oligo(dC)$_{30}$-C1S-V5, slow pathway. (**c**) Voltage dependence of the rate of step 4→1: (O) V5-C109-oligo(dC)$_{30}$; (Δ) oligo(dC)$_{30}$-C1S-V5. Each data point for the N-terminus-first measurements is derived from an exponential fit to data obtained from at least 3 different pores (n = 3).

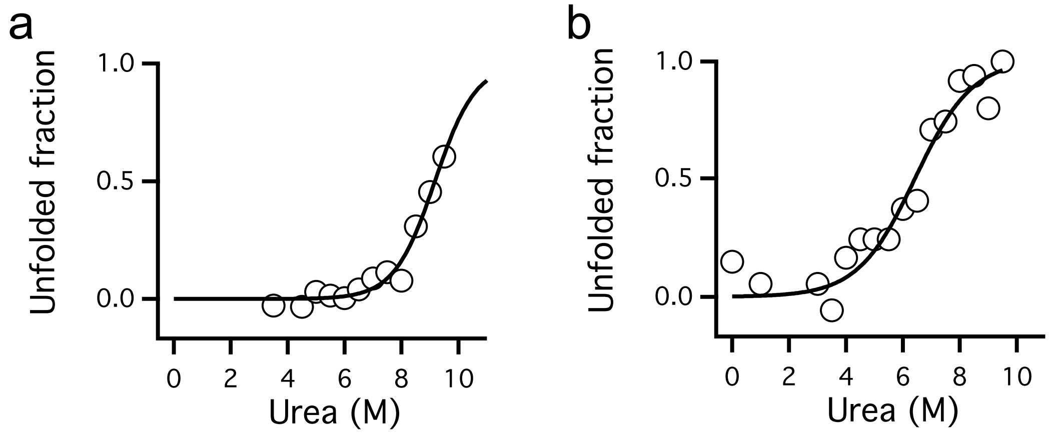

Supplementary Figure 2: Urea-unfolding curves measured by circular dichroism. (**a**) Trx S1C-V5, the solid line represents the fit to a two-state model (values from the fit are given in the main text). (**b**) Trx ▽6G-V5, the solid line represents the fit to a two-state model (values from the fit are given in the main text). Both experiments were carried out in 1 mM DTT, 2 M KCl, pH 7.2, 22 ºC. Ellipticity was measured at 222 nm.

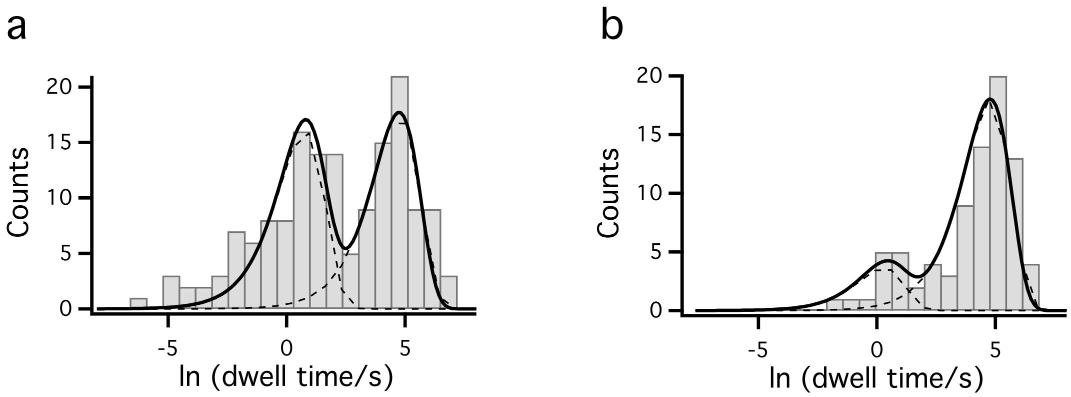

Supplementary Figure 3: Dwell time distributions in level 3 for N-terminus threading into the αHL pore. (**a**) For Trx oligo(dC)$_{30}$-S1C-V5, the two unfolding pathways are equally populated. (**b**) N-terminus threading of Trx oligo(dC)$_{30}$-P22A-V23I-V5. In this case, most of the Trx molecules go through the slow pathway for step 3→4. Both distributions were fitted to a double exponential function (black line). The dashed lines show the deconvolution into single exponentials. Data from at least 3 different pores were used to construct each histogram.

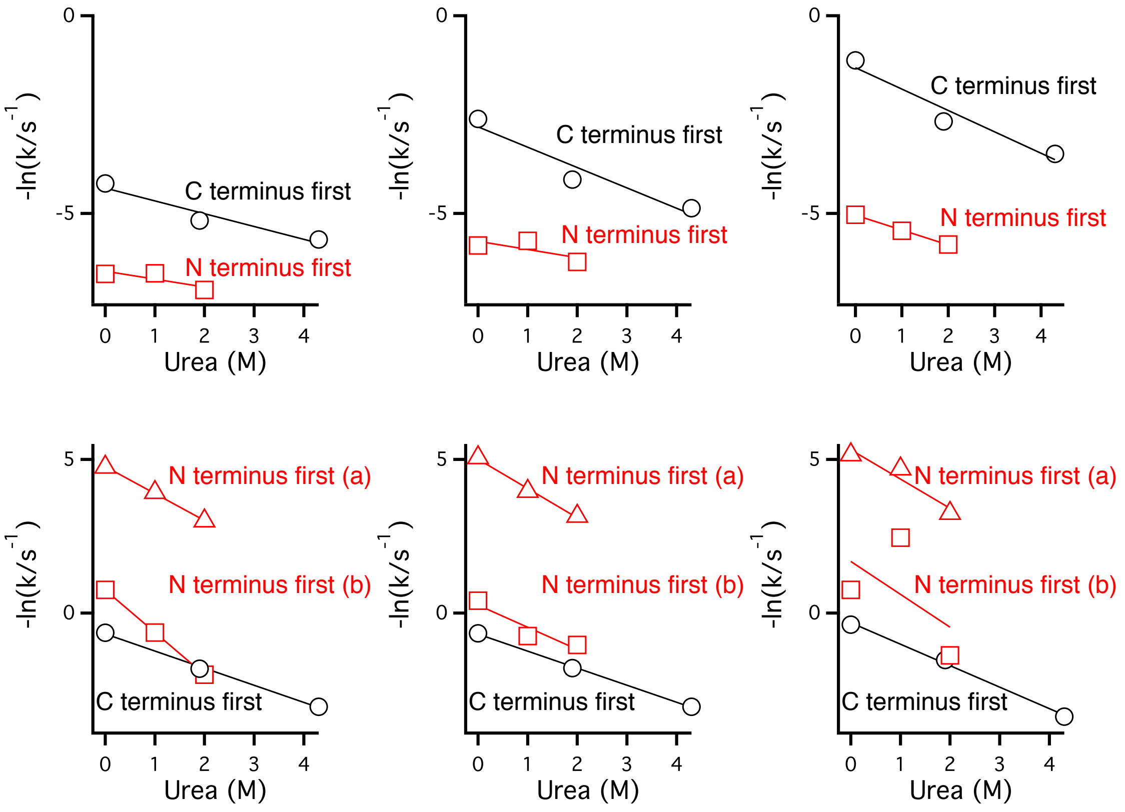

Supplementary Figure 4: Kinetics of steps 2→3 and 3→4 in the presence of urea. (**a**) Step 2→3, +140 mV. (**b**) Step 2→3, +120 mV. **(c)** Step 2→3, +100 mV. (**d**) Step 3→4, +140 mV. (**e**) Step 3→4, +120 mV. (**f**) Step 3→4, +100 mV. Each data set was fitted to a straight line and the average of slopes between voltages used to derive the kinetic m-values. Each point was obtained from data from at least 3 different pores.

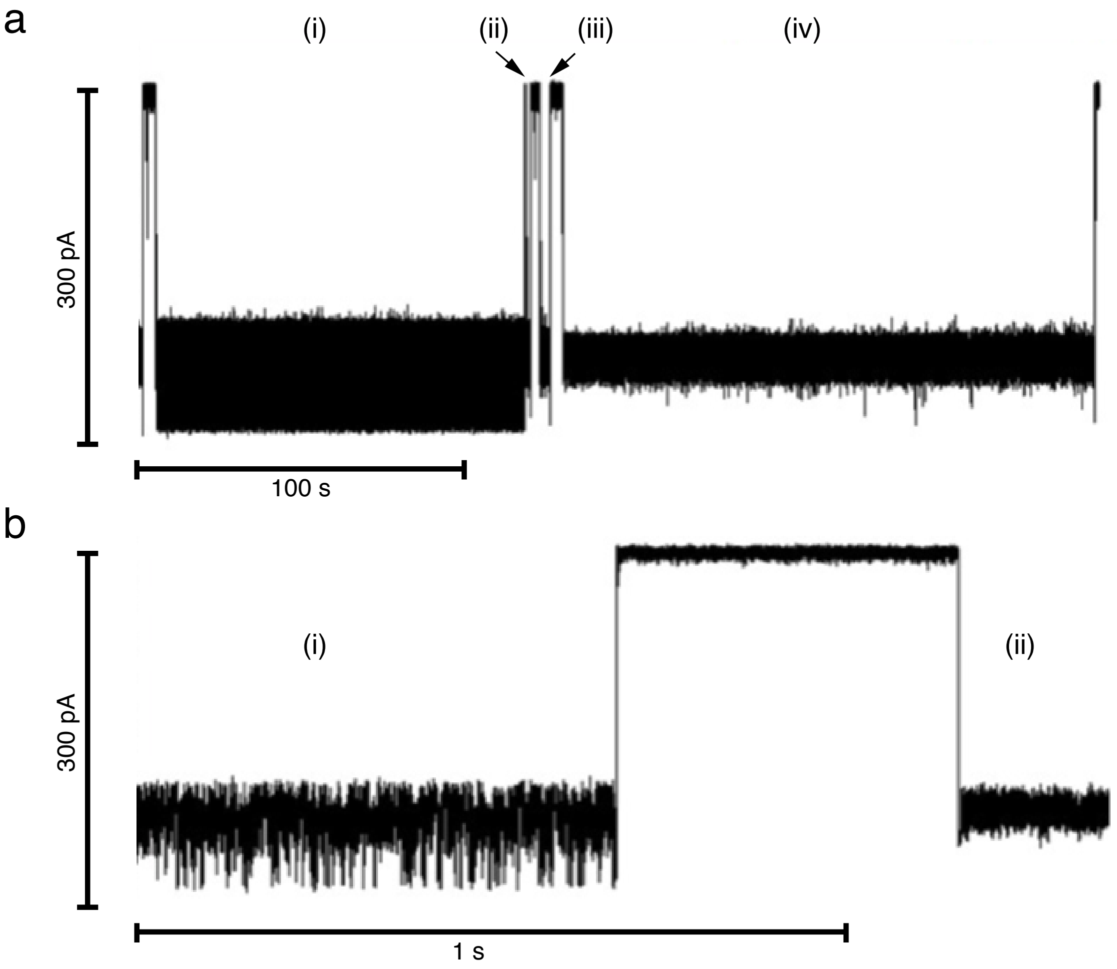

Supplementary Figure 5: Current measurements for the translocation of Trx oligo(dC)$_{30}$-S1C-V5 through the αHL pore (N-terminus first). (**a**) Four events are displayed: Event (i) has a total duration of >100 s and a noisy level 3. Events (ii) and (iii) have total durations of <5 s and normal levels 3. Event (iv) has a total duration of >100 s and a normal level 3. (**b**) Zoom showing the noisy level 3 of event (i). Events with a noisy level 3 represented 40% of the total number of events analysed (91% of the total: see the text). The events show similar kinetics, whether level 3 is noisy or not (Supplementary Fig. 6). Level 3 occasionally switched from a noisy state to the normal state, and vice versa. We attribute the differences in noise to different arrangements of the polypeptide when threaded within the pore.

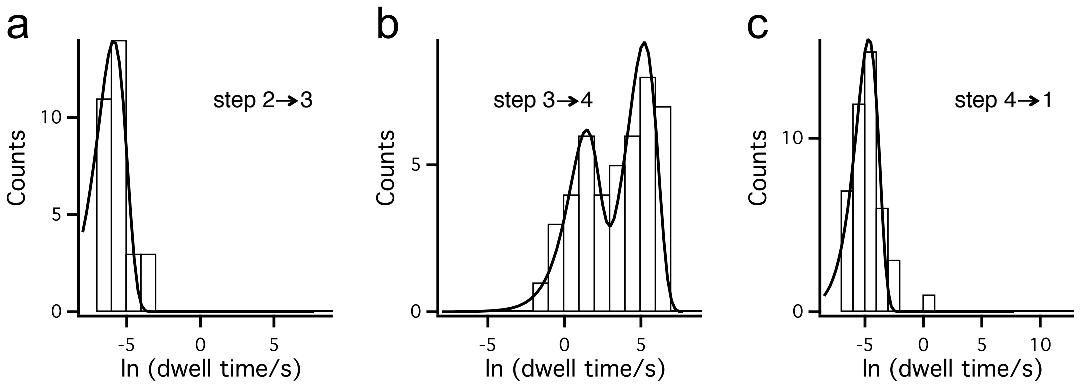

Figure 6: Dwell time distributions from events that show a noisy level 3 after N-terminus threading. (**a**) Histogram of dwell times in level 2 obtained at +140 mV. Solid line, fit to a single exponential distribution with rate $k_{2\rightarrow3}$ = 323 ± 17 s^{-1}. (**b**) Histogram of dwell times in level 3 obtained at +140 mV. Solid line, fit to a double exponential distribution with rates $k_{3a\rightarrow4}$ = 0.26 ± 0.03 s^{-1} and $k_{3b\rightarrow4}$ = 0.0054 ± 0.0003 s^{-1}. (**c**) Histogram of dwell times in level 4 obtained at +140 mV; solid line, fit to a single exponential distribution with rate $k_{4\rightarrow1}$ = 107 ± 6 s^{-1}. Data from at least 10 different pores were used to construct each histogram.

Supplementary Reference

1.- Rodriguez-Larrea, D. and Bayley, H. Multistep protein unfolding during nanopore translocation. *Nat. Nanotechnol*. **8**, 288-95 (2013).
